# Supplementary material for: Botrytis cinerea PMT4 Is Involved in O-Glycosylation, Cell Wall Organization, Membrane Integrity, and Virulence
Source: J Fungi (Basel). 2025 Jan 17;11(1):71. doi: 10.3390/jof11010071 (PMC11766925; doi:10.3390/jof11010071)
Supplement: Supplementary file 1 [file jof-11-00071-s001.zip › Caption Supplementary FigS1.pdf]

Supplementary Figures

Figure S1 Schematic representation of the *bcpmt4* disruption event. A. Schematic representation of the disruption event **B.** Diagnostic PCR analysis of genomic DNA isolated from WT and two independent *Δbcpmt4* mutants using primer pairs P1fw+P4rv (lane 1-3), Pro-fw +hphint-rev (lane 4), Pro-fw/hphint-rv and Hphin-fw+ter-rv (lane 5 and 6 respectively) **C.** RT-PCR analysis using primers P1fw/P2rv (lane 1, 2 and 3) and P3fw/P4rv (lane 4, 5 and 6) of the Δ*bcpmt4*/*bcpmt4* strain showed that was able to express *bcpmt4* gene. (Primers sequences are listed in Table S1).
