# Supplementary material for: Botrytis cinerea PMT4 Is Involved in O-Glycosylation, Cell Wall Organization, Membrane Integrity, and Virulence
Source: J Fungi (Basel). 2025 Jan 17;11(1):71. doi: 10.3390/jof11010071 (PMC11766925; doi:10.3390/jof11010071)
Supplement: Supplementary file 1 [file jof-11-00071-s001.zip › Table S1_v2.pdf]

Table. S1: Primers used in this study.

Name	Nucleotide sequence (5' - 3')	References
hph-fw	CACAGGATCCCTGCAGCTGTGGAGCCGCATTCC	[42]
hph-rv	TGTGGTCGACCGGCGCGCCGAGTTAGGCAACC	[42]
pmt4-P1fw	CACACCCGGGATGTCTTCTCAAGGTTCCGTTCG	This work
pmt4-P2rv	TGTGGGATCCGCCCCTAGAAGAGCAGGCATAGCG	This work
pmt4-P3fw	CACAGTCGACCGTGGAGTTGATGCATTAGATAACC	This work
pmt4-P4rv	TGTGGCATGCTTATTTGGCGAAATGAAGATCATATCC	This work
pmt4--Pro-fw	GTCAGTCAACACGAGAGACAAC	This work
pmt4-Ter-rv	GGTGACAGTATCATAGTTAGAC	This work
pmt4-Pattb1	GGGGACAAGTTTGTACAAAAAAGCAGGCTGCACTGTACCTTGAT	This work
pmt4-Pattb2	GGGGACCACTTTGTACAAGAAAGCTGGGTGCTAAAGTACCAGCG	This work
pmt4-Pex-fw	TATACCATGG ATGTCTTCTCAAGGTTCCGTTC	This work
pmt4-Pex-rv	TATAGGATCC TTATTTGGCGAAATGAAGATC	This work
