# Supplementary material for: Botrytis cinerea PMT4 Is Involved in O-Glycosylation, Cell Wall Organization, Membrane Integrity, and Virulence
Source: J Fungi (Basel). 2025 Jan 17;11(1):71. doi: 10.3390/jof11010071 (PMC11766925; doi:10.3390/jof11010071)
Supplement: Supplementary file 1 [file jof-11-00071-s001.zip › Table S2_v2.pdf]

Table S2. Estimated hygromycin copy number (HCN)" was determined by relative quantification qPCR. The mean fold change in expression of the target gene was calculated using ΔΔCT value.

**Strain**	**$\Delta C_{T\ sample}$***	**Calibrator***	**$\Delta\Delta C_T$***	**HCN**
				**$(1+E)^{-\Delta\Delta CT}$ ****
bcpmt4Δ	-0,99 ± 0,01	-0,62 ± 0,09	-0,37 ± 0,11	1,31
bcpmt4Δ/ bcpmt4	12,54 ± 0,07		13,15 ± 0,03	0,00

* average ± SD (n=3)

**relative amount of target by Livak and Schmittgen [46]
