# Supplementary figures and images for: Botrytis cinerea PMT4 Is Involved in O-Glycosylation, Cell Wall Organization, Membrane Integrity, and Virulence
Source: J Fungi (Basel). 2025 Jan 17;11(1):71. doi: 10.3390/jof11010071 (PMC11766925; doi:10.3390/jof11010071)

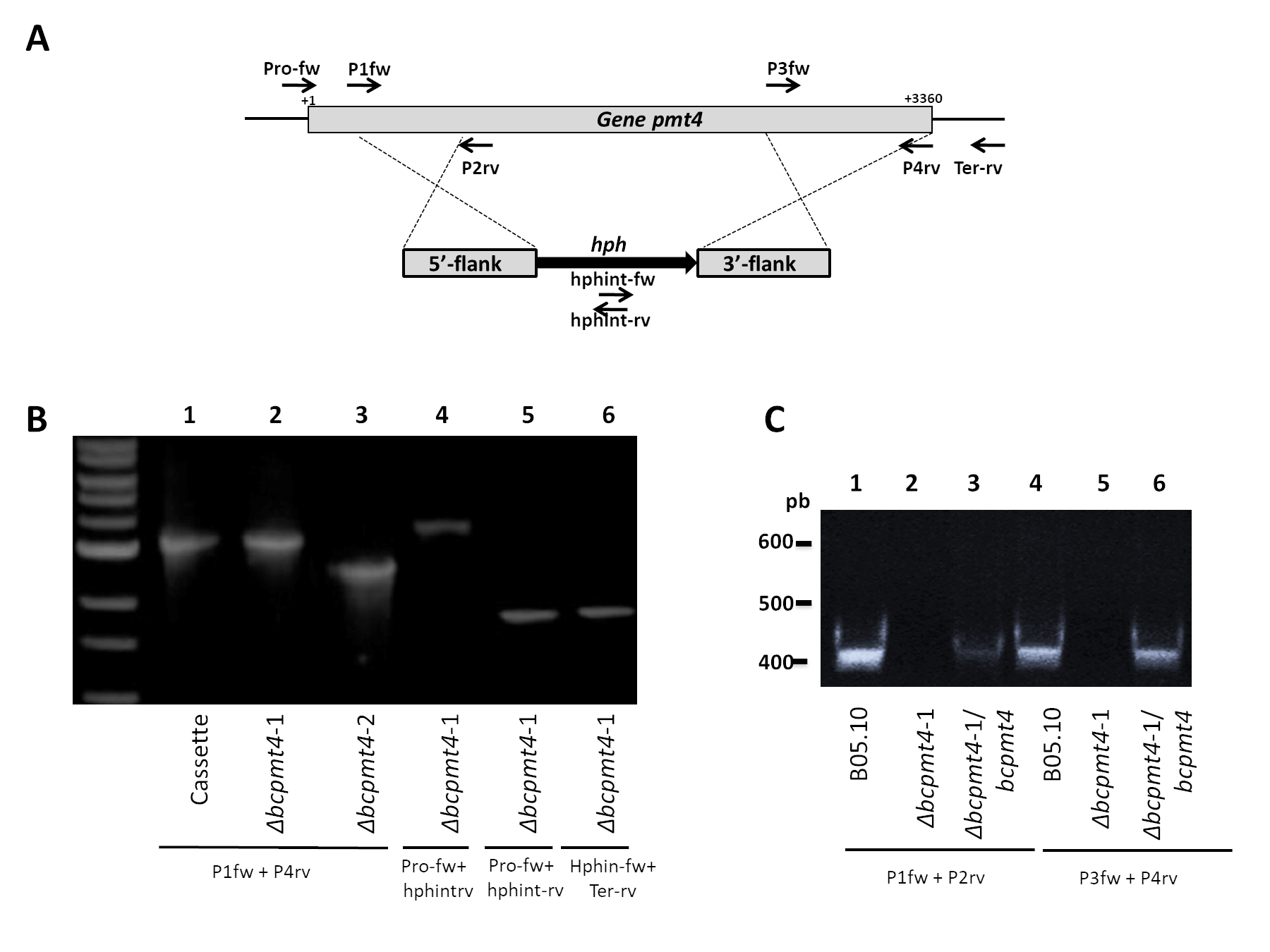

Supplement: Supplementary file 1 [file jof-11-00071-s001.zip › Fig S1.tif]
